# Supplementary material for: Reaction with ROO• and HOO• Radicals of Honokiol-Related Neolignan Antioxidants
Source: Molecules. 2023 Jan 11;28(2):735. doi: 10.3390/molecules28020735 (PMC9867055; doi:10.3390/molecules28020735)
Supplement: Supplementary file 1 [file molecules-28-00735-s001.zip › molecules-2101524-supplementary.pdf]

SUPPORTING INFORMATION

Reaction with ROO• and HOO• Radicals of Honokiol-Related Neolignan Antioxidants

Nunzio Cardullo [1], Filippo Monti [2], Vera Muccilli [1], Riccardo Amorati [3,*] and Andrea Baschieri [2,*]

[1] Dipartimento di Scienze Chimiche, Università di Catania, V.le A. Doria 6, 95125 Catania, Italy
[2] Istituto per la Sintesi Organica e la Fotoreattività (ISOF), Consiglio Nazionale delle Ricerche (CNR) Via Gobetti 101, 40129 Bologna, Italy
[3] Dipartimento di Chimica "G. Ciamician", Università di Bologna, Via S. Giacomo 11, 40126 Bologna, Italy
* Correspondence: riccardo.amorati@unibo.it (R.A.); andrea.baschieri@isof.cnr.it (A.B.)

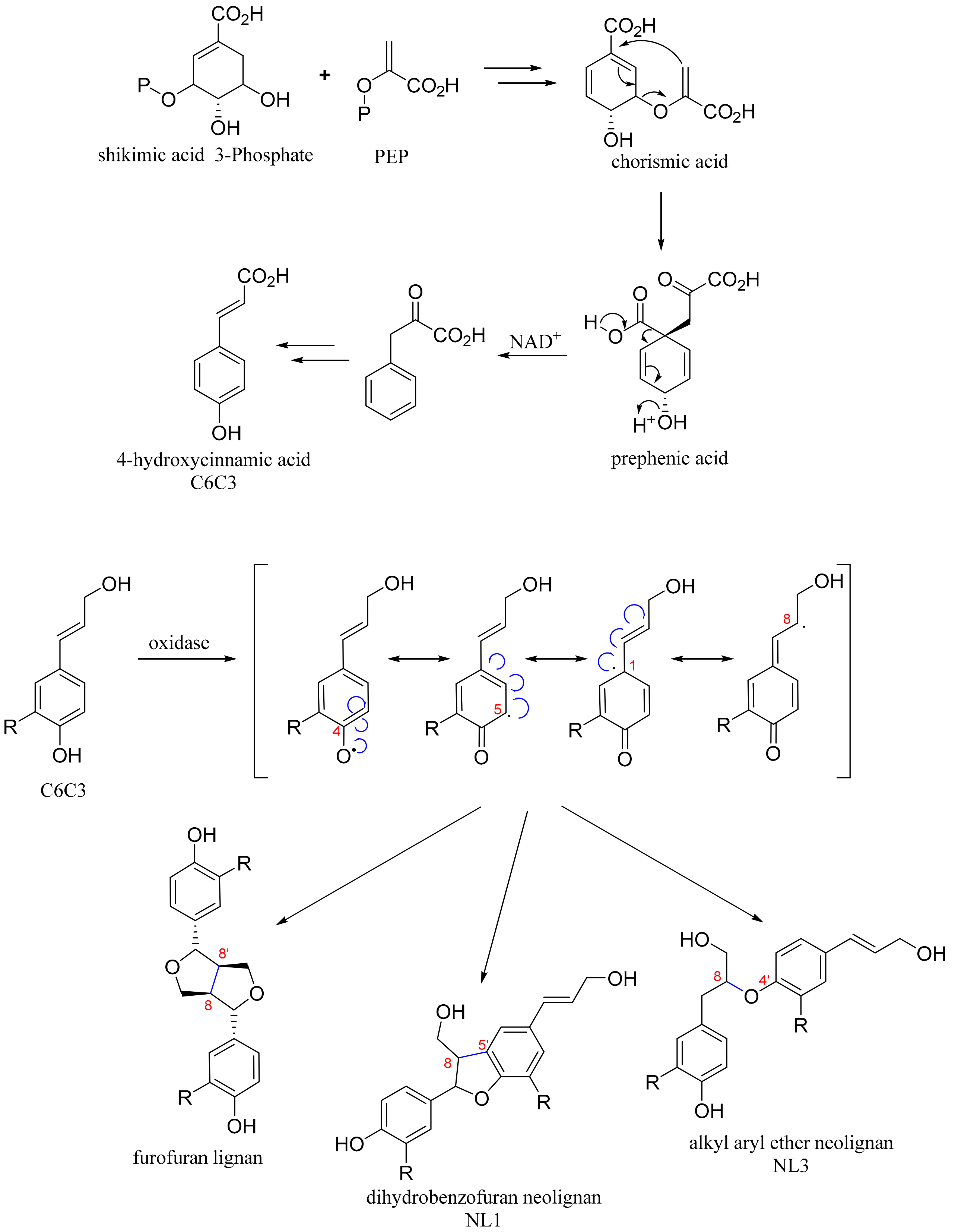

Figure S1. Schematization of biosynthetic pathway for the synthesis of phenylpropanoids C6C3, lignans and neolignans [56]. NL1 and NL3 are referred to neolignans according to the classifications of Teponno et al. [4].

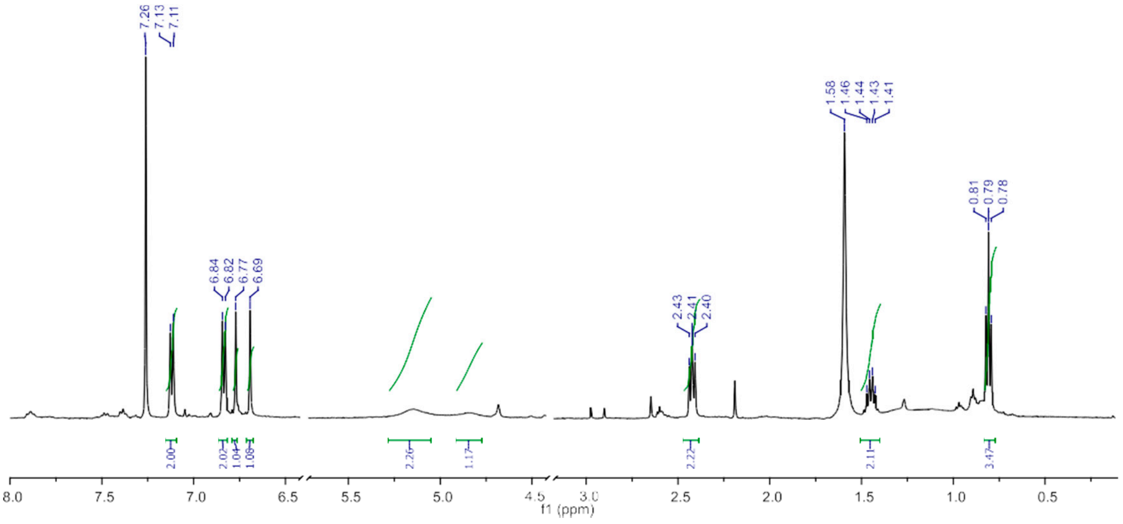

Figure S2. ^{1}H NMR spectrum (500 MHz, $CDCl_3$) of **7**.

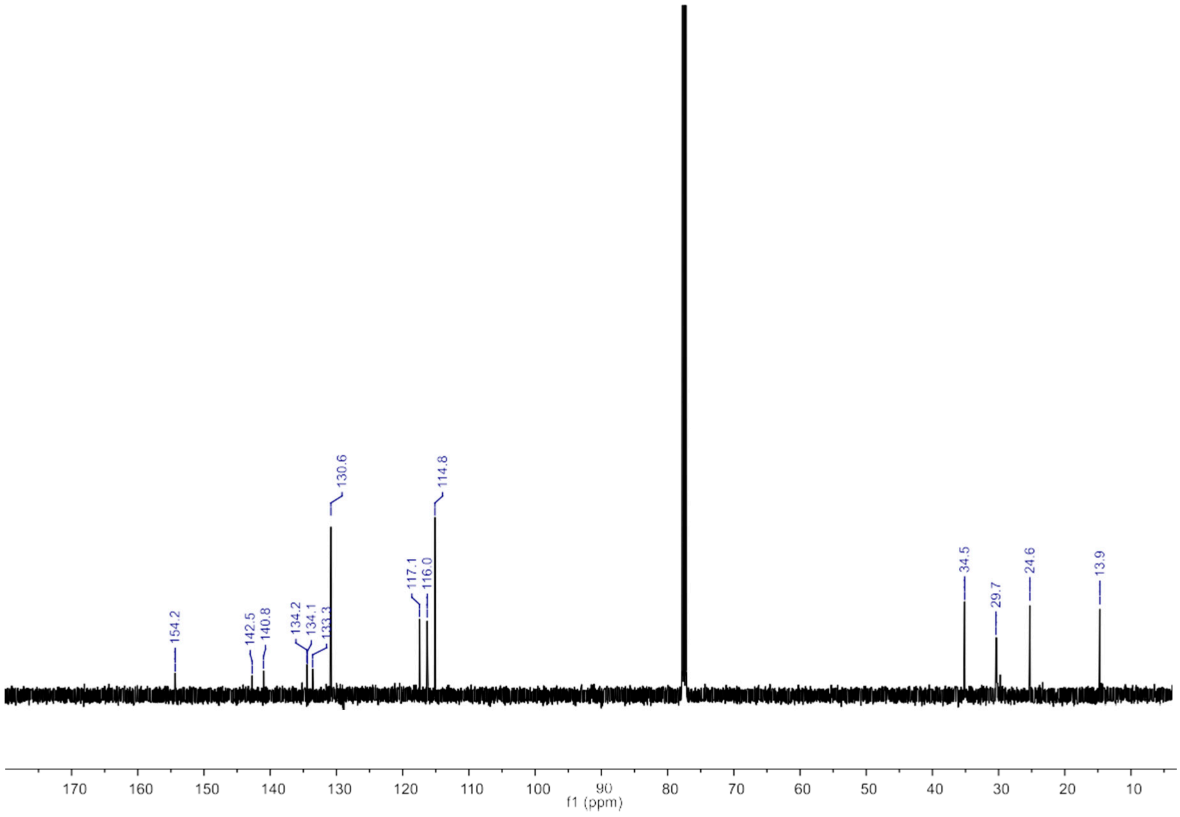

Figure S3. ^{13}C NMR spectrum (500 MHz, $CDCl_3$) of **7**.

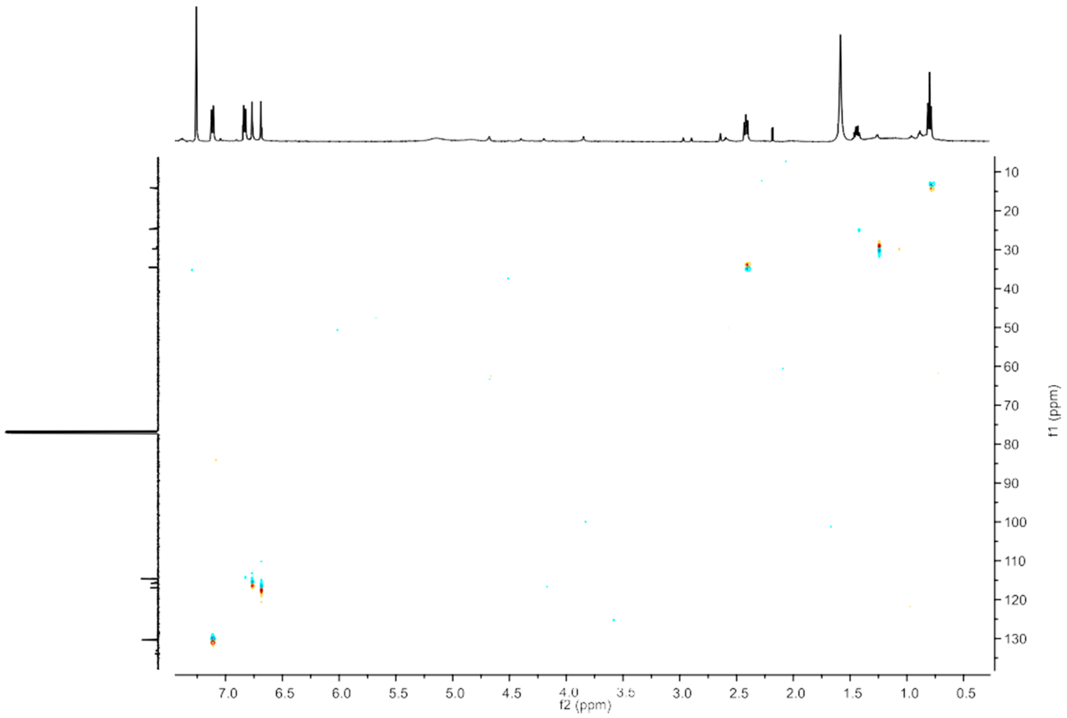

Figure S4. gHSQC spectrum of **7.**

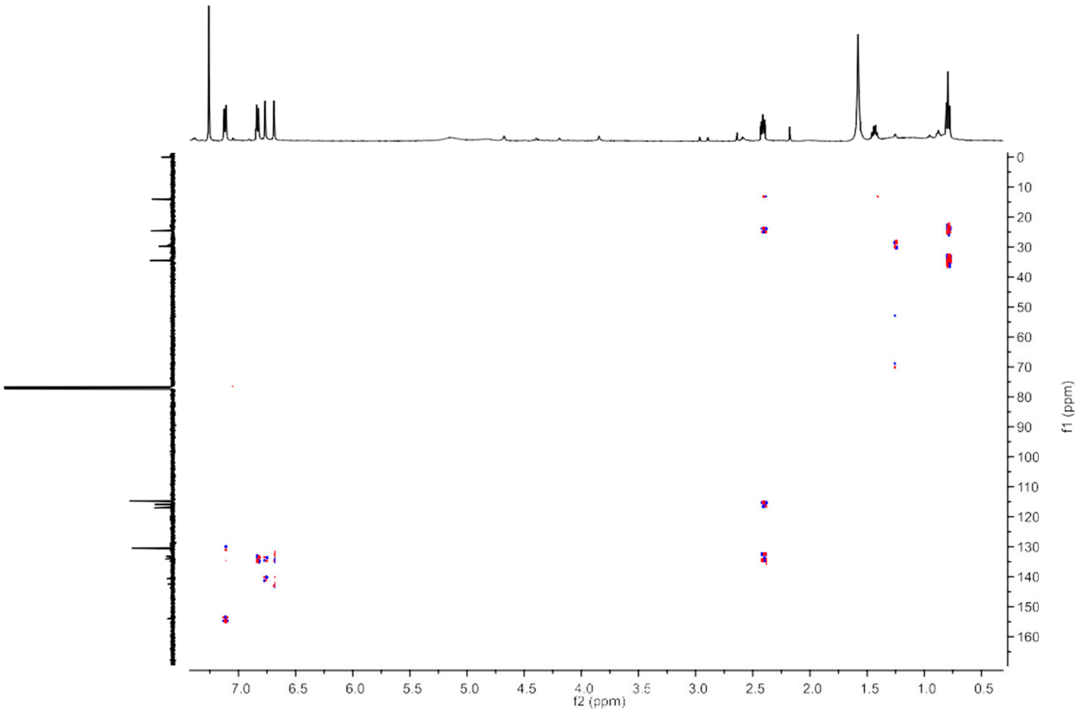

Figure S5. gHMBC spectrum of **7**.

References

4. Teponno, R.B.; Kusari, S.; Spiteller, M. Recent advances in research on lignans and neolignans. *Nat. Prod. Rep.* **2016**, *33*, 1044–1092.
56. Dewick, P.M. The shikimate pathway: aromatic amino acids and phenylpropanoids. In *Medicinal Natural Products: A Biosynthetic Approach*; 3rd ed.; John Wiley & Sons Ltd: Hoboken, NJ, USA, 2009; pp.137–186. https://doi.org/10.1002/9780470742761.ch4
